# Genomic confirmation of vancomycin-resistant *Enterococcus* transmission from deceased donor to liver transplant recipient

**DOI:** 10.1371/journal.pone.0170449

**Published:** 2017-03-16

**Authors:** Ali Bashir, Oliver Attie, Mitchell Sullivan, Robert Sebra, Kavindra V. Singh, Deena Altman, Theodore Pak, Jayeeta Dutta, Kieran Chacko, Elizabeth Webster, Martha Lewis, Camille Hamula, Kristin W. Delli Carpini, Barbara E. Murray, Andrew Kasarskis, Harm van Bakel, Shirish Huprikar

**Affiliations:** 1 Institute and Department of Genetics and Genomic Sciences, Icahn School of Medicine at Mount Sinai, New York, NY; 2 Division of Infectious Diseases, Department of Medicine, The University of Texas Medical School, Houston, TX; 3 Division of Infectious Diseases, Department of Medicine, Icahn School of Medicine at Mount Sinai, New York, NY; 4 Department of Pathology, Icahn School of Medicine, New York, NY; 5 LiveOnNY, New York, NY; Leibniz-Institute DSMZ, GERMANY

## Abstract

In a liver transplant recipient with vancomycin-resistant *Enterococcus* (VRE) surgical site and bloodstream infection, a combination of pulsed-field gel electrophoresis, multilocus sequence typing, and whole genome sequencing identified that donor and recipient VRE isolates were highly similar when compared to time-matched hospital isolates. Comparison of *de novo* assembled isolate genomes was highly suggestive of transplant transmission rather than hospital-acquired transmission and also identified subtle internal rearrangements between donor and recipient missed by other genomic approaches. Given the improved resolution, whole-genome assembly of pathogen genomes is likely to become an essential tool for investigation of potential organ transplant transmissions.

## Introduction

Donor-derived bacterial infections are a recognized early complication of solid organ transplantation (SOT)[1]. The current definition of transmission requires “clear evidence of the same infection in the donor and at least one of the recipients.”[1] However, common hospital-acquired bacteria such as methicillin-resistant *Staphylococcus aureus* (MRSA) and vancomycin-resistant *Enterococcus* (VRE) could infect the donor and recipient independently, and it may be difficult to distinguish donor acquisition from hospital acquisition. This is particularly true when infection is not immediately recognized in the recipient. Automated *de novo* construction of high-quality bacterial genomes using long-read whole genome sequencing (WGS) is a powerful tool that can aid in donor transmission epidemiology[2–5]. In our previous work, WGS approaches were used to confirm donor transmission of MRSA to a liver transplant recipient[6]. Here, we employ multiple genomics strategies, culminating in WGS, to demonstrate donor acquisition of VRE in a liver transplant recipient with surgical site and bloodstream infection by comparing donor and recipient genomes with contemporary hospital isolates.

## Case report

### Donor

A 51-year-old woman was hospitalized for an exacerbation of systemic lupus erythematosus. The hospital course was complicated by respiratory failure from diffuse alveolar hemorrhage, acute kidney injury requiring renal replacement therapy, and ultimately irreversible central nervous system damage. She was pronounced brain dead and her liver was recovered for transplant. All cultures were negative at this time.

### Recipient

A 65-year-old man with hepatitis C cirrhosis and hepatocellular carcinoma was admitted from home and underwent deceased donor liver transplantation. There were no significant intra-operative complications except for expected blood product requirements. The post-operative course was complicated by the development of a complex fluid collection that gradually increased in size; ongoing need for blood transfusion; and acute kidney injury requiring continuous veno-venous hemofiltration (CVVH). Renal function improved and CVVH was discontinued on post-operative day 6. Fever developed immediately after discontinuation of CVVH. Blood cultures obtained on post-operative days 7–9 grew VRE. Linezolid was started on post-operative day 10. The patient was taken to the operating room on post-operative day 13, and one liter of hematoma was evacuated. Cultures of the hematoma grew VRE. Follow-up blood cultures were negative and fever resolved. Linezolid was discontinued thirteen days after evacuation of the hematoma.

Donor blood cultures at the donor hospital eventually grew Gram-positive cocci in pairs and chains and yeast on post-transplant days 1 and 2, respectively. These culture results were finalized on post-transplant day 6 as VRE and *Candida glabrata*, respectively. The final donor culture results were communicated to our hospital on post-transplant day 15. Caspofungin was started in the recipient at this time and continued for 21 days; however, none of the recipient cultures grew *C*. *glabrata*. The recipient was eventually discharged home after a 49-day hospitalization. He has not required any further hospitalizations and is doing well approximately five years later with excellent graft function.

## Materials and methods

The donor blood VRE isolate (VRE Donor) was obtained from the donor hospital by LiveOnNY (formerly New York Organ Donor Network) for comparison with the recipient blood isolate (VRE Recipient). Ten additional VRE blood culture isolate strains in the preceding eight weeks from the recipient hospital were retrieved for comparison.

### PFGE

VRE Donor, VRE Recipient and three control (VRE 5, 6, 7) isolates were selected for pulse-field gel electrophoresis (PFGE). Agarose plugs containing genomic DNA were digested with SmaI, and pulsed-field gel electrophoresis (PFGE) was performed using a previously described method[7], but with ramped pulse times of 2 s and 28 s to resolve higher-molecular weight fragments and 2 s and 7s s to resolve low-molecular weight fragment**s**.

### PacBio DNA preparation and sequencing

For whole genome sequencing, single bacterial colonies were grown overnight in Luria-Bertani (LB) broth and high molecular weight DNA extraction was performed as previously described[6]. DNA library preparation and Pacific Biosciences (PacBio) sequencing was performed according to the manufacturer’s instructions and reflects the P5-C3 sequencing enzyme and chemistry, respectively. For each isolate, large insert libraries size-selected for fragments greater than 7kb were run on the PacBio RS. A detailed description of the sequencing protocol can be found in S1 File.

#### Genome assembly, circularizitation, resequencing, annotation

PacBio sequences were assembled using HGAP3 (https://github.com/PacificBiosciences/SMRT-Analysis/wiki/SMRT-Analysis-Software-Installation-v2.2.0). Assemblies were circularized using a custom pipeline employing Nucmer[8]. In short, contig ends that show strong overlap at the boundaries (overlapping alignments exceeding 500 bp) were suggestive of a circular joining site. To eliminate overlapping sequences at the end of contigs and to increase accuracy, the assemblies were joined at the implied overlap point, reoriented (to cause the ends to be internal sequences), and these circularized assemblies were re-sequenced using SMRTpipe 2.2.0. Illumina sequences were mapped to the polished assemblies using bwa-mem[9] (version 0.7.10) in order to verify SNPs and correct assembly errors. Isolates were sequenced to sufficient coverage for *de novo* assembly on the PacBio RS II. The resulting assemblies were utilized for multilocus sequence typing (MLST) and whole-genome comparative analyses. Table A in S1 File shows sequencing input and assembly quality for all isolates.

#### Illumina resequencing and variant resolution

In order to further improve assembly quality Illumina resequencing was performed for nine isolates, including VRE Donor and Recipient. Briefly, 0.5–1 μg of input DNA taken from the same aliquot used for Pacbio sequencing was sheared to an average fragment size of 200 bp on a Bioruptor Pico sonicator (Diagenode). Next, amplicon sequence libraries were prepared using the end repair, A-tailing, and adaptor ligation NEBNext DNA library prep modules for Illumina from New England Biolabs according to the manufacturer’s protocol. Following final size-selection with 1x volume Ampure XP beads, and secondary PCR (8 cycles) to introduce barcoded primers, multiplexed libraries were sequenced on the Illumina HiSeq 2500 platform in a single-end 100nt run format.

Sequence errors in the assemblies were corrected by mapping Illumina reads to the assembled contigs with BWA mem[10]. Variants were called using bcftools[11] and then corrected in the assembly. In repetive regions where one repeat contains more errors than the other, BWA mem will map all reads to the copy of the repeat with the least errors. This results in the more erroneous region remaining uncorrected. In order to correct these regions, reads were remapped to sections of the genome with significantly lower than normal coveage (half the median). Indels in these errors were then corrected if a variant was called with bcftools with more than 90% of reads in consensus.

To help resolve potentially missed plasmids, Illumina reads were also assembled using SPAdes[12] version 3.6.0 with default settings. Assembled contigs were then aligned to the PacBio assembly using BLAST+[13]. Illumina contigs that mapped along more than 90% of their length at 90% or greater identity were removed as they are considered to be part of the original PacBio + Illumina assembly. Next, low-coverage non-bacterial contaminant sequences were removed and remaining contigs were circularized using Circlator[14] and annotated by Prokka[15] to identify small plasmids < 10kb (Table B in S1 File). For the donor and recipient genomes complete, circularized assemblies were created by including contigs generated from both the illumina and PacBio assemblies. NCBI accession numbers for deposited assemblies are contained in Table C in S1 File.

#### MLST and vancoymcin typing

MLST typing was performed by uploading whole genome assemblies to http://pubmlst.org, a public database of MLST types, which includes data for *Enterococcus faecium* and by using core genome MLST (cgMLST) as part of Ridom SeqSphere+ (http://www.ridom.com/seqsphere/cgmlst/). The pubMLST database returned an MLST type for each genome and types for each allele in seven genes: adk, atpA, ddl, gdh, gyd, pstS and purK; these types were confirmed by SeqSphere for all strains except VRE1, for which no MLST type was given (Table D in S1 File). A subset (10) genomes were further verified using PCR and Sanger sequencing with 9/10 confirming the predicted MLST type obtained from WGS. The only differing strain type was VRE11; Sanger sequencing of VRE11 differed in the atpA and ddl gene assignments which yielded an MLST type that has not previously been observed (potentially an error in the Sanger result).

To determine the vancomycin resistance genotype, PacBio amino acid sequences were analyzed with CARD’s RGI software (3.0.9) using default settings[16]. Initial results indicated an incomplete van operon in VRE 11 missing vanHA and vanXA, respectively. Results were therefore cross-referenced with Illumina nucleotide sequences which identified a complete vancomycin resistance operon. Initial results also indicated vanB ligase genes occurring in some genomes. All CARD reported vancomycin genes were run through BLAST to confirm vanA ligase identity. All isolates were tested with Vitek; genotypes and MIC values are provided in Table D in S1 File.

#### Comparison between strains and phylogenetic analysis

**Fig 1:**
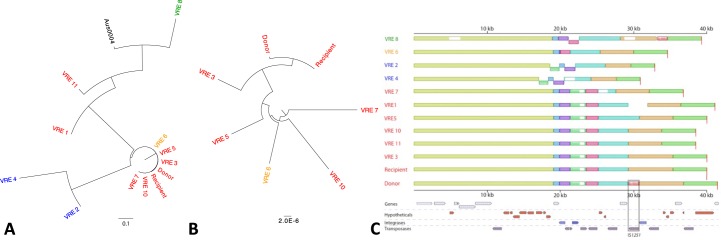
Genomic comparison of VRE isolates. **(A)** Conserved sequence blocks generated by HarvestTools 1.1.2 to construct the phylogenetic tree. **(B)** A second phylogeny was performed on more closely related strains, to refine the Recipient-Donor clade. Isolates with the hypervariable groupings shown in **(C)** share the same color in both phylogenies. **(C)** A 40kb interval from the donor strain spanning the hypervariable chromosomal locus was extracted and homologous sequence blocks were obtained from each isolate. Each colored block corresponds to syntenic interval between strains, and isolates are grouped by primary syntenic block order. The red recipient block (outlined in black) corresponds to a mobile element gene insertion of an IS*1251*-like element. The corresponding genes in the Recipient genomic interval are shown below.

Phylogenetic analyses was performed using HarvestTools v1.1.2[17]. First Parsnp v1.2 was used to obtained core genomes from our Pacbio assemblies, utilizing the Aus0004 genome as reference[18] and filtering for PhiPack identified regions of recombination[19]. Prokka[20] was used for genome annotations and Roary v3.6.1[21] was used to calculate core gene set sizes. *De novo* assemblies were also to examined for potential structural differences via Nucmer alignment (MUMmer version 3.23)[8]. Lastly, Progressive Mauve[22] was used to create the multiple sequence alignments shown in **Fig 1B**.

## Results

### PFGE

**Fig 2:**
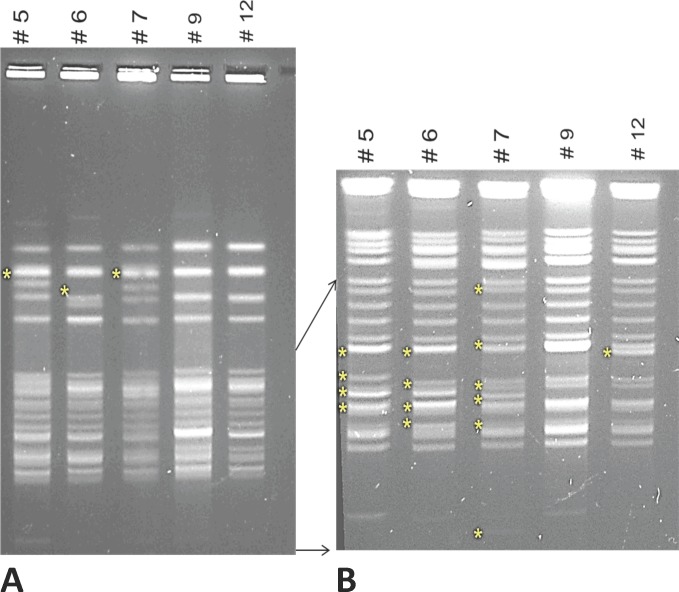
Agarose gel showing SmaI digestion patterns of strains via PFGE. **(A)** PFGE gel of VRE 5, 6, 7, VRE Recipient and VRE Donor isolates with ramped pulse times of 2s to 28s to resolve higher-molecular weight fragments. **(B)** Same as A but with ramped pulse times of 2s to 7s to resolve low-molecular weight fragments. Asterisks (*) indicate missing or variable DNA band sizes among isolates. Arrows indicate the corresponding higher resolution DNA band area in panel B relative to panel A.

VRE Donor and VRE Recipient had almost identical SmaI restriction enzyme PFGE patterns, differing only in the presence of a single brighter band (**Fig 2**). The control strains were considerably different, both from each other and from VRE Donor and VRE Recipient, showing greater than 6 band changes (**Fig 2**) following Tenover et al. criteria[23].

### MLST

While VRE Donor and Recipient had the same ST736 MLST genotype, this genotype was shared with three other control isolates from the recipient hospital: VRE 5, VRE 6, and VRE 7. Nearly all remaining isolates represented distinct MLSTs with genotypes that have been identified worldwide and seem to be widely distributed[24–29].

### Whole genome comparison

The *de novo* assembled VRE genomes were highly contiguous; in all cases, the assemblies contained fewer than 10 contigs with the largest contig representing more than 50% of the total genome length. The isolates were quite diverse, containing a core genome of 2161 genes with an average of 3140 genes per genome. **Fig 1A** shows a phylogenetic tree representing SNP distances between the 12 VRE strains based on whole genome alignment (Methods). The resulting phylogeny identified a Donor-Recipient specific subclade within the ST736 MLST subtype. Pairwise whole genome comparison was then performed between VRE Donor and VRE Recipient which were assembled to completion. While the two isolates were nearly identical at the SNV level, containing a single SNV within the main chromosome, they were not completely structurally identical: Four intervals exhibited inserted or deleted sequence between the isolates (Figure A and Table E in S1 File), and a 1.5kb plasmid interval contained 15 intergenic SNVs. All of the inserted or deleted sequences contained tranposases with similarity to known insertion sequences (ISs), IS*1251*[30] and IS*Efa11*[31], previously associated with the *vanA* gene cluster. The insertion with high similarity to IS*1251* was missing from two locations (one chromosomal and one plasmid) within the VRE Donor. The corresponding chromosomal location was interrogated across all 12 isolates by isolating a 40kb flanking interval that encodes 29 genes including 10 mobile elements. **Fig 1B** and Table F in S1 File highlight the mobile insertion between donor and recipient. Notably, the orthologous region was structurally variable across nearly all isolates. Even those genomes with similar organization and block size, such as VRE 10 and VRE 11, contained multiple SNVs between one another. Based on ordering and directionality of syntenic blocks, these strains were separated into four categories (colors of isolates in **Fig 1A**); however, local variation (such as insertions and deletions and SNVs) still existed within each group. Closest strain pairs on the SNV phylogeny did not necessarily correspond to the closest structural matches for this region (e.g. VRE10 and VRE11), and in some cases large-scale rearrangements had occurred between two proximal isolates (e.g. VRE11 and VRE8). Together, this suggests that this region is likely to mutate or undergo rearrangement over a short period (as in the case of VRE Donor and VRE Recipient).

## Discussion

VRE surgical site and bloodstream infection in a hospitalized liver transplant recipient meets the definition of a hospital-acquired infection[32], but early bacterial infection may also be donor-transmitted. In our case, the possibility of donor-transmitted infection was only considered after the donor infection history was eventually obtained. By genomic analysis of VRE blood isolates from the donor, recipient, and control isolates from the recipient hospital, we were able to demonstrate that this is a likely case of donor transmission rather than hospital acquisition.

Our combination of short and long read genomic approaches highlights the increased specificity of WGS for resolving both SNV and structural differences between similar isolates. All genomic analyses revealed a strong donor and recipient grouping; however, multiple isolates had MLST types consistent with the donor and recipient. And, while PFGE did show a tighter grouping between donor and recipient, only the full *de novo* assembly was able to clarify the unique structural differences between the donor and recipient isolate. Interestingly, this and related insertion elements have previously been associated with increased antibiotic resistance in the context of the vanA gene cluster[33]. This type of transposase and mobile element variation is consistent with previous studies which have suggested that recombinational exchange is five times more likely to generate new alleles in *E*. *faecium*[34]. The high-rates of recombination seen in *E*. *faecium* have been shown in simulation to render MLST phylogenetic inference inaccurate[35]. In fact, large structural changes could also cause substantial band shifts or amplitude changes in PFGE profiles. Our data suggest that WGS may be increasingly necessary to unambiguously confirm transmission for structurally mutable genomes. Previous transmission studies define allowed numbers of mutations between two patients over a given time window based on molecular clocks[36], but such analyses are typically restricted to SNVs. In part, this is due to the ubiquity of short-read sequencing which are ideal for reference-based mapping but do not easily facilitate discovery of large scale structural variants insertions and rearrangements. As long-read sequencing becomes more readily available, and automated assembly algorithms continue to improve, it will become increasingly necessary to fold-in large-scale structural changes into genomic models of transmission.

In summary, WGS provided substantial evidence that VRE infection in our liver transplant recipient was not hospital acquired as would have typically been considered but rather transmitted from the deceased donor. We expect that WGS and assembly of pathogen genomes will be increasingly important not only for understanding pathogen biology and evolution but also become a routine and essential tool for investigation of potential organ transplant transmissions in many settings. Our case also highlights the importance of communicating deceased donor culture information to transplant recipient centers so that potential preventive or preemptive strategies can be implemented as early as possible.

## Supporting information

S1 File Supplemental Methods, Tables, and Figures. (DOCX)

## Abbreviations

VRE: vancomycin-resistant *Enterococcus*
WGS: whole genome sequencing
MLST: multilocus sequence typing
PFGE: pulsed-field gel electrophoresis
SOT: solid organ transplantation
MRSA: methicillin-resistant *Staphylococcus aureus*
CVVH: continuous veno-venous hemofiltration
SNV: single nucleotide variation
