# Supplementary material for: Genomic confirmation of vancomycin-resistant Enterococcus transmission from deceased donor to liver transplant recipient
Source: PLoS One. 2017 Mar 16;12(3):e0170449. doi: 10.1371/journal.pone.0170449 (PMC5354240; doi:10.1371/journal.pone.0170449)
Supplement: S1 File — (DOCX) [file pone.0170449.s001.docx]

**Supplemental Methods**

*SMRT sequencing of VRE isolates*

DNA library preparation and sequencing was performed according to the manufacturer’s instructions and reflects the P5-C3 sequencing enzyme and chemistry, respectively. In short, 5 µg of extracted, high-quality, genomic DNA from each of twelve VRE isolates was verified using Qubit analysis to quantify the mass of double-stranded DNA present. After quantification, each sample was diluted to 150 µL using Qiagen elution buffer at 33 µg / µL. The 150 µL aliquots were individually pipetted into the top chambers of Covaris G-tube spin columns and sheared gently for 60 seconds at 4500 rpm using an Eppendorf 5424 benchtop centrifuge and repeated at 60 seconds at 4500 rpm to further shear the DNA and place the aliquot back into the upper chamber, resulting in a ~20,000 bp DNA shear, verified using a DNA 12000 Agilent Bioanalyzer gel chip. The sheared DNA isolates were then re-purified using a 0.45X AMPure XP purification step (0.45X AMPure beads added, by volume, to each DNA sample dissolved in 200 µL EB, vortexed for 10 minutes at 2,000 rpm, followed by two washes with 70% alcohol and finally diluted in EB). There was no evidence of plasmids below 10kb, thus only one large insert, size-selected library was constructed.

After purification, 2.1 to 3.4 µg of each purified and sheared sample was taken into DNA damage and end-repair. Briefly, the DNA fragments were repaired using DNA Damage Repair solution (1X DNA Damage Repair Buffer, 1X NAD+, 1 mM ATP high, 0.1 mM dNTP, and 1X DNA Damage Repair Mix) with a volume of 21.1 µL and incubated at 37ºC for 20 minutes. DNA ends were repaired next by adding 1X End Repair Mix to the solution, which was incubated at 25ºC for 5 minutes followed by the second 0.45X Ampure XP purification step. Next, 0.75 µM of Blunt Adapter was added to the DNA followed by 1X template Prep Buffer, 0.05 mM ATP low and 0.75 U/µL T4 ligase to ligate (final volume of 47.5 µL) the SMRTbell adapters to the DNA fragments. This solution was incubated at 25ºC overnight followed by a 65ºC 10-minute ligase denaturation step. After ligation, the library was treated with an exonuclease cocktail to remove un-ligated DNA fragments using a solution of 1.81 U/µL Exo III 18 and 0.18 U/µL Exo VII, then incubated at 37ºC for 1 hour. Two additional 0.45X Ampure XP purifications steps were performed to remove <2000 bp molecular weight DNA and organic contaminant.

Upon completion of library construction, samples were validated as ~20 kb using another Agilent DNA 12000 gel chip. All libraries were sufficient for additional size selection to remove any library molecules <7,000 bp. This step was conducted using Sage Science Blue Pippin 0.75% agarose cassettes to select library in the range of 7,000-50,000 bp. This selection is necessary to narrow the library distribution and maximize the SMRTbell sub-readlength for the best *de novo* assembly possible. 11% to 27% of the input library eluted from the agarose cassette and was available for sequencing. This yield was sufficient to proceed to primer annealing and DNA sequencing on the PacBio RSII machine. Size-selection was confirmed by Bio-Analysis and the mass was quantified using the aforementioned Qubit assay.

Primer was then annealed to the size-selected SMRTbell with the full-length libraries (80ºC for 2 minute 30 seconds followed by decreasing the temperature by 0.1º to 25Cº). The polymerase-template complex was then bound to the P4 enzyme using a ratio of 10:1 polymerase to SMRTbell at 0.5 nM for 4 hours at 30ºC and then held at 4ºC until ready for magbead loading, prior to sequencing. The magnetic bead-loading step was conducted at 4ºC for 60-minutes per manufacturer’s guidelines. The magbead-loaded, polymerase-bound, SMRTbell libraries were placed onto the RSII machine at a sequencing concentration of 50 pM and configured for a 180-minute continuous sequencing run. Sequencing was conducted to ample coverage across four SMRTcells for each isolate. Data was then generated and assembled using the HGAP3 SMRTportal assembly pipeline, filtering at 0.80 RQ, 500 bp sub-readlength, and standard pre-assembly pipeline parameters[^1^](#_ENREF_1).

**Supplemental Tables**

**Table A: Sequencing Data Summary**

| Strain | Mapped Reads | PacBio Depth | Unmapped Reads | Total Reads | N50 | Contigs | Sum of Contig Lengths | Illumina Depth |
| --- | --- | --- | --- | --- | --- | --- | --- | --- |
| VRE1 | 129,418 | 218.34 | 13,564 | 142,982 | 3,130,373 | 6 | 3,259,287 | NA |
| VRE2 | 57,492 | 104.29 | 6,189 | 63,681 | 2,932,050 | 4 | 3,285,246 | 302.73 |
| VRE3 | 77,252 | 176.04 | 14,239 | 91,491 | 2,852,748 | 7 | 3,242,787 | 242.61 |
| VRE4 | 73,449 | 83.73 | 10,376 | 83,825 | 2,933,574 | 7 | 3,223,114 | 200.99 |
| VRE5 | 75,740 | 181.81 | 8,171 | 83,911 | 2,818,673 | 6 | 3,161,203 | 215.44 |
| VRE6 | 96,684 | 71.11 | 22,112 | 118,796 | 1,037,453 | 7 | 3,089,946 | NA |
| VRE7 | 81,226 | 144.76 | 6,252 | 87,478 | 2,850,245 | 5 | 3,221,356 | 227.10 |
| VRE8 | 65,720 | 117.20 | 7,934 | 73,654 | 2,970,757 | 6 | 3,366,191 | 271.31 |
| Recipient | 159,954 | 278.48 | 25,568 | 185,522 | 2,802,604 | 5 | 3,126,703 | 196.40 |
| VRE10 | 61,680 | 116.03 | 7,156 | 68,836 | 2,869,701 | 4 | 3,203,859 | 223.08 |
| VRE11 | 35,576 | 38.90 | 2,925 | 38,501 | 2,823,158 | 7 | 3,140,297 | NA |
| Donor | 71,781 | 140.00 | 11,468 | 83,249 | 2,807,999 | 5 | 3,130,591 | 191.57 |

**Table B: Genome Plasmid Summary**

| **Strain** | **Contig** | **Length** | **Circularized** |
| --- | --- | --- | --- |
| Donor | Main Chromosome | 2,807,999 | Yes |
| Donor | Plasmid p1 | 259,837 | Yes |
| Donor | Plasmid p2 | 45,240 | Yes |
| Donor | Plasmid p3 | 13,141 | Yes |
| Donor | Plasmid p4* | 4,374 | Yes |
| Recipient | Main Chromosome | 2,802,605 | Yes |
| Recipient | Plasmid p1 | 259,837 | Yes |
| Recipient | Plasmid p2 | 46,747 | Yes |
| Recipient | Plasmid p3 | 13,141 | Yes |
| Recipient | Plasmid p4* | 4,374 | Yes |
| VRE 1 | Main chromosome | 3,109,381 | Yes |
| VRE 1 | Plasmid p1 | 44,226 | Yes |
| VRE 1 | Plasmid p2 | 57,543 | No |
| VRE 1 | Plasmid p3 | 13,123 | Yes |
| VRE 1 | Plasmid p4 | 13,136 | No |
| VRE 10 | Main chromosome | 2,869,701 | No |
| VRE 10 | Plasmid p1 | 215,250 | Yes |
| VRE 10 | Plasmid p2 | 78,475 | No |
| VRE 10 | Plasmid p3 | 40,433 | No |
| VRE 10 | Plasmid p4* | 11,589 | No |
| VRE 10 | Plasmid p5* | 6,663 | No |
| VRE 10 | Plasmid p6* | 3,023 | No |
| VRE 10 | Plasmid p7* | 5,982 | No |
| VRE 11 | Main chromosome | 2,823,158 | No |
| VRE 11 | Plasmid p1 | 202,822 | No |
| VRE 11 | Plasmid p2 | 48,256 | Yes |
| VRE 11 | Plasmid p3 | 832 | No |
| VRE 11 | Plasmid p5 | 58,308 | No |
| VRE 11 | Plasmid p6 | 2,125 | No |
| VRE 11 | Plasmid p7 | 4,796 | No |
| VRE 2 | Main chromosome | 2,932,067 | Yes |
| VRE 2 | Plasmid p1 | 196,671 | No |
| VRE 2 | Plasmid p10* | 2,713 | No |
| VRE 2 | Plasmid p11* | 3,630 | No |
| VRE 2 | Plasmid p2 | 81,539 | No |
| VRE 2 | Plasmid p3 | 74,986 | No |
| VRE 2 | Plasmid p4* | 4,899 | No |
| VRE 2 | Plasmid p5* | 1,959 | No |
| VRE 2 | Plasmid p6* | 1,725 | No |
| VRE 2 | Plasmid p7* | 2,464 | No |
| VRE 2 | Plasmid p8* | 2,244 | No |
| VRE 2 | Plasmid p9* | 4,768 | No |
| VRE 3 | Main chromosome | 2,852,748 | Yes |
| VRE 3 | Plasmid p1 | 201,621 | No |
| VRE 3 | Plasmid p10* | 1,639 | No |
| VRE 3 | Plasmid p11* | 651 | No |
| VRE 3 | Plasmid p12* | 1,797 | No |
| VRE 3 | Plasmid p13* | 3,798 | No |
| VRE 3 | Plasmid p14* | 4,321 | No |
| VRE 3 | Plasmid p15* | 759 | No |
| VRE 3 | Plasmid p16* | 865 | No |
| VRE 3 | Plasmid p2 | 12,140 | No |
| VRE 3 | Plasmid p3 | 57,676 | Yes |
| VRE 3 | Plasmid p4 | 33,769 | Yes |
| VRE 3 | Plasmid p5 | 71,684 | No |
| VRE 3 | Plasmid p6 | 13,149 | No |
| VRE 3 | Plasmid p7* | 4,375 | No |
| VRE 3 | Plasmid p8* | 6,428 | No |
| VRE 3 | Plasmid p9* | 1,074 | No |
| VRE 4 | Main chromosome | 2,933,574 | Yes |
| VRE 4 | Plasmid p1 | 182,290 | No |
| VRE 4 | Plasmid p2 | 56,686 | No |
| VRE 4 | Plasmid p3 | 6,809 | No |
| VRE 4 | Plasmid p4 | 39,395 | Yes |
| VRE 4 | Plasmid p5 | 5,250 | No |
| VRE 4 | Plasmid p6 | 9,312 | Yes |
| VRE 4 | Plasmid p7* | 4,429 | No |
| VRE 4 | Plasmid p8* | 3,022 | No |
| VRE 5 | Main chromosome | 2,818,673 | Yes |
| VRE 5 | Plasmid p1 | 205,001 | Yes |
| VRE 5 | Plasmid p2 | 54,823 | Yes |
| VRE 5 | Plasmid p3 | 10,075 | No |
| VRE 5 | Plasmid p4 | 40,072 | Yes |
| VRE 5 | Plasmid p5 | 31,926 | No |
| VRE 5 | Plasmid p6* | 3023 | No |
| VRE 5 | Plasmid p7* | 4385 | No |
| VRE 6 | Main chromosome | 1,069,964 | No |
| VRE 6 | Main chromosome | 1,037,453 | No |
| VRE 6 | Main chromosome | 515,594 | No |
| VRE 6 | Main chromosome | 141,070 | No |
| VRE 6 | Main chromosome | 55,914 | No |
| VRE 6 | Plasmid p1 | 205,921 | Yes |
| VRE 6 | Plasmid p2 | 64,030 | Yes |
| VRE 7 | Main chromosome | 2,850,245 | Yes |
| VRE 7 | Plasmid p1 | 200,151 | Yes |
| VRE 7 | Plasmid p2 | 77,569 | No |
| VRE 7 | Plasmid p3 | 54,805 | Yes |
| VRE 7 | Plasmid p4 | 38,586 | No |
| VRE 7 | Plasmid p5* | 2,415 | No |
| VRE 7 | Plasmid p6* | 4,891 | No |
| VRE 8 | Main chromosome | 2,970,757 | No |
| VRE 8 | Plasmid p1 | 105,869 | No |
| VRE 8 | Plasmid p1 | 105,869 | No |
| VRE 8 | Plasmid p10* | 3,772 | No |
| VRE 8 | Plasmid p11* | 3,110 | No |
| VRE 8 | Plasmid p12* | 869 | No |
| VRE 8 | Plasmid p13* | 1,066 | No |
| VRE 8 | Plasmid p14* | 4,921 | No |
| VRE 8 | Plasmid p15* | 1,371 | No |
| VRE 8 | Plasmid p2 | 210,514 | No |
| VRE 8 | Plasmid p3 | 84,196 | No |
| VRE 8 | Plasmid p4 | 49,389 | Yes |
| VRE 8 | Plasmid p5 | 3,036 | No |
| VRE 8 | Plasmid p6* | 3,014 | No |
| VRE 8 | Plasmid p7* | 1,005 | No |
| VRE 8 | Plasmid p8* | 4,284 | No |
| VRE 8 | Plasmid p9* | 2,353 | No |

***Assembled from Illumina data**

(Note: Any non-circularized fragments may be split among multiple chromosome/plasmid ids)

**Table C: Accession Numbers for Sequencing Data and Assemblies**

| Isolate | Type of Data | Accession |
| --- | --- | --- |
| VRE 1 | Assembly | CP012430-CP012435 |
| VRE 2 | Assembly | CP012436-CP012439 |
| VRE 3 | Assembly | CP012440-CP012446 |
| VRE 4 | Assembly | CP012447-CP012453 |
| VRE 5 | Assembly | CP012454-CP012459 |
| VRE 6 | Assembly | LIVR00000000 |
| VRE 7 | Assembly | CP012460-CP012464 |
| VRE 8 | Assembly | CP012465-CP012470 |
| Recipient | Assembly | CP018825-CP018829 |
| VRE 10 | Assembly | CP012471-CP012474 |
| VRE 11 | Assembly | LIHD00000000 |
| Donor | Assembly | CP018830-CP018834 |
| VRE 2 | Illumina Reads | SRR3115443 |
| VRE 2 | Illumina Reads | SRX1543354 |
| VRE 3 | Illumina Reads | SRR3115446 |
| VRE 3 | Illumina Reads | SRX1543363 |
| VRE 4 | Illumina Reads | SRR3115450 |
| VRE 4 | Illumina Reads | SRX1543364 |
| VRE 5 | Illumina Reads | SRR3115453 |
| VRE 5 | Illumina Reads | SRX1543365 |
| VRE 7 | Illumina Reads | SRR3115512 |
| VRE 7 | Illumina Reads | SRX1543420 |
| VRE 8 | Illumina Reads | SRR3115534 |
| VRE 8 | Illumina Reads | SRX1543443 |
| Recipient | Illumina Reads | SRR3115540 |
| Recipient | Illumina Reads | SRX1543444 |
| VRE 10 | Illumina Reads | SRR3115312 |
| VRE 10 | Illumina Reads | SRX1543246 |
| Donor | Illumina Reads | SRR3115319 |
| Donor | Illumina Reads | SRX1543251 |
| VRE 1 | PacBio Reads | SRX1258250 |
| VRE 2 | PacBio Reads | SRX1541965 |
| VRE 3 | PacBio Reads | SRX1542020 |
| VRE 4 | PacBio Reads | SRX1542021 |
| VRE 5 | PacBio Reads | SRX1542071 |
| VRE 6 | PacBio Reads | SRX1542072 |
| VRE 7 | PacBio Reads | SRX1542107 |
| VRE 8 | PacBio Reads | SRX1542146 |
| Recipient | PacBio Reads | SRX1542110 |
| VRE 10 | PacBio Reads | SRX1542146 |
| VRE 11 | PacBio Reads | SRX1542424 |
| Donor | PacBio Reads | SRX1542425 |

**Table D: MLST Types , MICs and Sources for VRE isolates**

| **Isolate** | ***adk*** | ***atpA*** | ***ddl*** | ***gdh*** | ***gyd*** | ***pstS*** | ***purK*** | **Type** | **cgMLST type** | **Vancomycin MIC** | **Source** | **Vancomycin Genotype** |
| --- | --- | --- | --- | --- | --- | --- | --- | --- | --- | --- | --- | --- |
| **VRE1** | N/A | 7 | 1 | 1 | 5 | 1 | 1 | 18 | ? | >32 | Clinical/ Blood | vanA |
| **VRE2** | 1 | 15 | 3 | 1 | 1 | 20 | 44 | 656 | 656 | 256 | Clinical/ Blood | vanA |
| **VRE3** | 1 | 1 | 3 | 1 | 1 | 1 | 44 | 736 | 736 | >32 | Clinical/ Blood | vanA |
| **VRE4** | 1 | 15 | 1 | 1 | 1 | 20 | 44 | 412 | 412 | >32 | Clinical/ Blood | vanA |
| **VRE5** | 1 | 1 | 3 | 1 | 1 | 1 | 44 | 736 | 736 | >32 | Clinical/ Blood | vanA |
| **VRE6** | 1 | 1 | 3 | 1 | 1 | 1 | 44 | 736 | 736 | >32 | Clinical/ Blood | vanA |
| **VRE7** | 1 | 1 | 3 | 1 | 1 | 1 | 44 | 736 | 736 | >32 | Clinical/ Blood | vanA |
| **VRE8** | 1 | 1 | 1 | 1 | 1 | 1 | 1 | 17 | 17 | >32 | Clinical/ Blood | vanA |
| **Recipient** | 1 | 1 | 3 | 1 | 1 | 1 | 44 | 736 | 736 | >32 | Clinical/ Blood | vanA |
| **VRE10** | 1 | 1 | 3 | 1 | 1 | 1 | 44 | 736 | 736 | >32 | Clinical/ Blood | vanA |
| **VRE11** | 1 | 7 | 1 | 1 | 5 | 1 | 1 | 18 | 18 | >32 | Clinical/ Blood | vanA |
| **Donor** | 1 | 1 | 3 | 1 | 1 | 1 | 44 | 736 | 736 | >16 | Clinical/ Blood | vanA |

**Table D: Genomic differences between Donor and Recipient**

| Chromosome/Plasmid | Type of change | Position (in donor isolate) | Size | Genes affected |
| --- | --- | --- | --- | --- |
| Chromosome | SNV | 2282333 | 1 | Nonsynonymous change to *yycG* |
| Chromosome | Insertion in donor isolate | 1073721 | 5334 | Duplication of transposase, insertion of 2 hypothetical proteins with sequence similar to a putative membrane protein and conserved virulence factor B |
| Chromosome | Deletion in donor isolate | 1791203 | 1507 | Deletion of region containing transposase |
| Chromosome | Duplication in donor isolate | 2509417 | 1568 | Duplication of region containing transposase |
| Plasmid p1 | Variable region | 2613..4090 | 1477 | 15 base changes between 2613..4090, all in intergenic region |
| Plasmid p2 | Deletion in donor isolate | 12126 | 1507 | Deletion of putative insertion element |

**Table F: Potential Coding Sequences in Figure 1B**

**(Coordinates relative to the start of the Recipient Genomic Interval)**

| **Start** | **End** | **Strand** | **Annotation** |
| --- | --- | --- | --- |
| 608 | 2689 | + | gene=cadA;product=Cadmium%2C zinc and cobalt-transporting ATPase |
| 3000 | 4466 | + | gene=ycaM;product=Inner membrane transporter YcaM |
| 5081 | 5620 | + | product=topology modulation protein |
| 5694 | 6134 | + | gene=copY;product=Transcriptional repressor CopY |
| 6147 | 6356 | + | gene=copZ;product=Copper chaperone CopZ |
| 6356 | 8542 | + | gene=copA;product=putative copper-importing P-type ATPase A |
| 8562 | 10733 | + | gene=copB;product=Copper-exporting P-type ATPase B |
| 10907 | 12124 | - | product=Transposase |
| 12367 | 13107 | - | product=Transglutaminase-like superfamily protein |
| 13240 | 13575 | - | product=hypothetical protein |
| 13672 | 14538 | - | product=hypothetical protein |
| 14528 | 15469 | - | product=hypothetical protein |
| 15614 | 16462 | - | product=ATPase family associated with various cellular activities (AAA) |
| 16816 | 17628 | + | product=hypothetical protein |
| 17782 | 18105 | + | product=hypothetical protein |
| 18179 | 18748 | + | product=hypothetical protein |
| 19185 | 19877 | + | gene=lytA;product=Autolysin |
| 19917 | 20723 | - | product=Integrase core domain protein |
| 20720 | 21265 | - | product=Transposase |
| 21361 | 21651 | + | product=Transposase |
| 21687 | 22523 | + | product=Integrase core domain protein |
| 22825 | 23643 | + | product=Transposase DDE domain protein |
| 23895 | 25073 | - | product=Transposase%2C Mutator family |
| 25327 | 25743 | + | product=hypothetical protein |
| 25975 | 26232 | - | product=hypothetical protein |
| 26518 | 27768 | - | product=Transposase IS116/IS110/IS902 family protein |
| 28178 | 29152 | + | gene=cbh;product=Choloylglycine hydrolase |
| **29341** | **30633** | **-** | **product=Transposase (Missing from Donor)** |
| 30782 | 31741 | - | product=Integrase core domain protein |
| 32103 | 33398 | - | product=Transposase |
| 34005 | 34322 | + | product=hypothetical protein |
| 34413 | 34778 | - | product=hypothetical protein |
| 34986 | 35468 | + | product=hypothetical protein |
| 35665 | 36555 | + | gene=pstS1;product=Phosphate-binding protein PstS 1 precursor |
| 36677 | 36835 | - | product=hypothetical protein |
| 36842 | 38116 | - | product=Transposase |
| 38389 | 40860 | + | product=Calcium-transporting ATPase |
| 40963 | 41493 | - | gene=senX3;product=Signal-transduction histidine kinase senX3 |

**Supplemental Figures:**

**
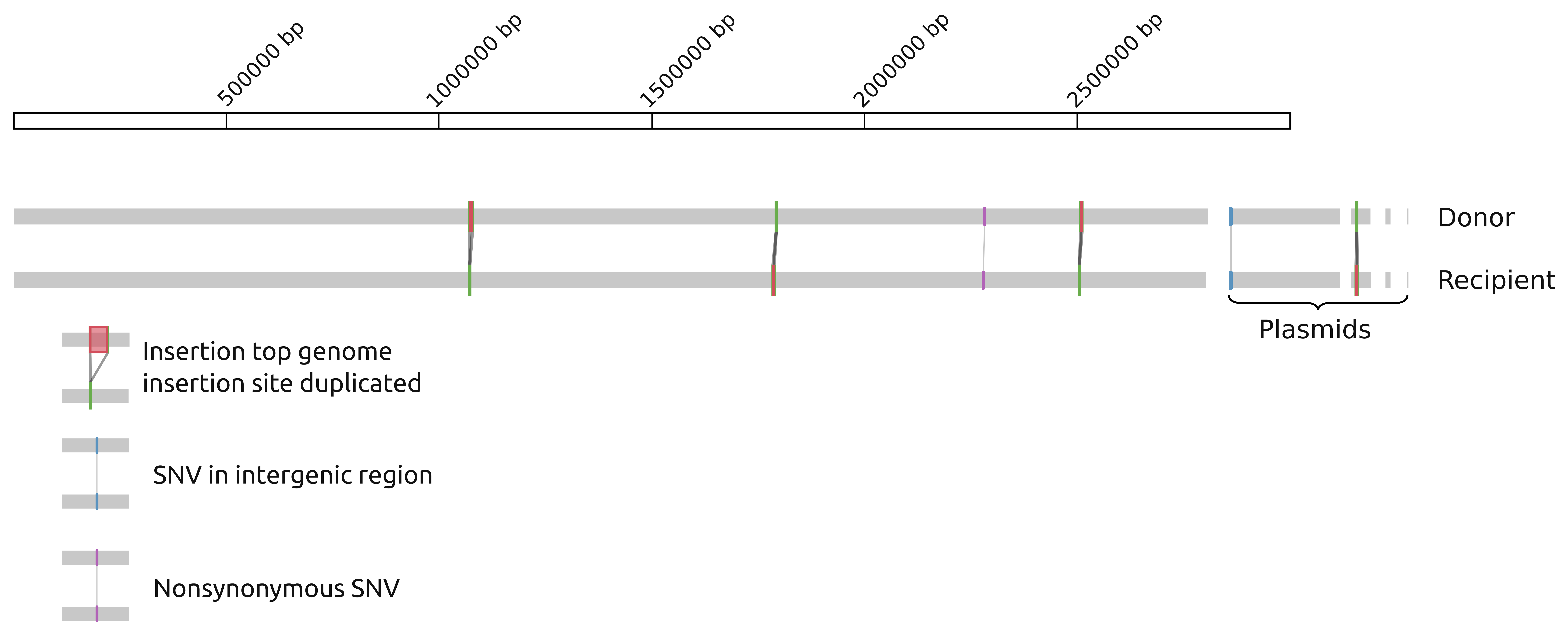
**

**Figure A: Genomic changes between donor and recipient isolates.** Illustration of all changes between donor and recipient isolates. Each chromosome or plasmid is represented by a gray, horizontal rectangle. Structural variations are represented by colored rectangles with a width equal to the size of the variant. SNVs are represented by colored lines.
